# The genome sequence of the Common Darter, *Sympetrum striolatum *(Charpentier, 1840)

**DOI:** 10.12688/wellcomeopenres.19937.1

**Published:** 2023-09-01

**Authors:** Liam M. Crowley, Benjamin W. Price, E. Louise Allan, Marianne Eagles, University of Oxford and Wytham Woods Genome Acquisition Lab, Natural History Museum Genome Acquisition Lab, Darwin Tree of Life Barcoding collective, Wellcome Sanger Institute Tree of Life programme, Wellcome Sanger Institute Scientific Operations: DNA Pipelines collective, Tree of Life Core Informatics collective, Darwin Tree of Life Consortium

**Affiliations:** 1 University of Oxford, Oxford, England, UK; 2 Natural History Museum, London, England, UK; 3 Independent researcher, Crawley Down, England, UK

**Keywords:** Sympetrum striolatum, Common Darter, genome sequence, chromosomal, Odonata

## Abstract

We present a genome assembly from an individual female *Sympetrum striolatum* (the Common Darter; Arthropoda; Insecta; Odonata; Libellulidae). The genome sequence is 1349.6 megabases in span. Most of the assembly is scaffolded into 12 chromosomal pseudomolecules, including the X sex chromosome. The mitochondrial genome has also been assembled and is 16.16 kilobases in length.

## Species taxonomy

Eukaryota; Metazoa; Eumetazoa; Bilateria; Protostomia; Ecdysozoa; Panarthropoda; Arthropoda; Mandibulata; Pancrustacea; Hexapoda; Insecta; Dicondylia; Pterygota; Palaeoptera; Odonata; Epiprocta; Anisoptera; Cavilabiata; Libellulidae; *Sympetrum*; *Sympetrum striolatum* (Charpentier, 1840) (NCBI:txid6969).

## Background

*Sympetrum striolatum,* or Common Darter, of the Libellulidae family is a common and abundant Eurasian dragonfly, widespread across Europe, Britain and Ireland ( Dijkstra *et al.*, 2020; GBIF Secretariat, 2022). It is listed as ‘Least Concern’ on the International Union for Conservation of Nature red list status ( IUCN, 2022). The flight season in the UK and across northern Europe is from June to November, while in southern Europe it may be seen all year round. The Common Darter prefers warm stagnant water, and is found in a variety of habitats, including ponds, lakes, ditches, canals, slow rivers, and brackish water ( Smallshire & Swash, 2004).

*Sympetrum* species are difficult to distinguish from one another, and the Common Darter can be mistaken for the Ruddy Darter ( *S. sanguineum*). The cream or yellow stripes on the outer sides of their legs are a distinguishing feature of *S. striolatum*. Yellow panels on the sides of the thorax in males are also a characteristic of the Common Darter. Males have an orange-red abdomen, while females and immature individuals have a yellow to brown abdomen. Examination of the male and female genitalia also assists identification of different species ( Brooks & Cham, 2014).

Combining morphological and molecular data increases the accuracy of species identification. The Highland Darter *Sympetrum nigrescens*, once distinguished as a separate species, is now not recognised as an independent species due to the sharing of CO1 haplotypes with *Sympetrum striolatum* ( Pilgrim & Von Dohlen, 2007). It has been proposed that *S. nigrescen*s may be a melanistic variant linked to its more northern and colder habitats ( Dijkstra *et al.*, 2020). Feng *et al*. (2020) described the sequencing and annotating of the complete mitogenome of a *S. striolatum* specimen from the Altay Region in China. The mitogenome was used to produce a mitochondrial phylogeny of 29 Odonata species, which supported the monophyly of Libellulidae and revealed that *S. striolatum* is closely related to the Asian species *Brachythemis contaminata* ( Feng *et al.*, 2020).

The availability of a whole genome sequence will deepen our understanding of this dragonfly. We present a chromosomally complete genome sequence for *Sympetrum striolatum*, based on one female specimen from Wytham Woods, Oxfordshire, as part of the Darwin Tree of Life Project. This project is a collaborative effort to sequence all named eukaryotic species in the Atlantic Archipelago of Britain and Ireland.

## Genome sequence report

**Figure 1.**
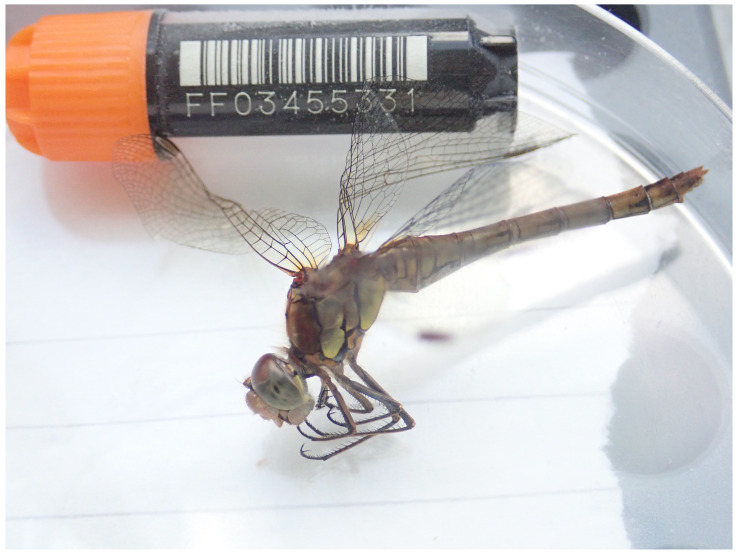
Photograph of the *Sympetrum striolatum* (ioSymStri1) specimen used for genome sequencing.

The genome was sequenced from one female *Sympetrum striolatum* ( Figure 1) collected from Wytham Woods, Oxfordshire, UK (51.77, –1.33). A total of 40-fold coverage in Pacific Biosciences single-molecule HiFi long reads and 24-fold coverage in 10X Genomics read clouds were generated. Primary assembly contigs were scaffolded with chromosome conformation Hi-C data. Manual assembly curation corrected 56 missing joins or mis-joins and removed 40 haplotypic duplications, reducing the assembly length by 2.72% and the scaffold number by 11.41%, and increasing the scaffold N50 by 7.86%.

**Figure 2.**
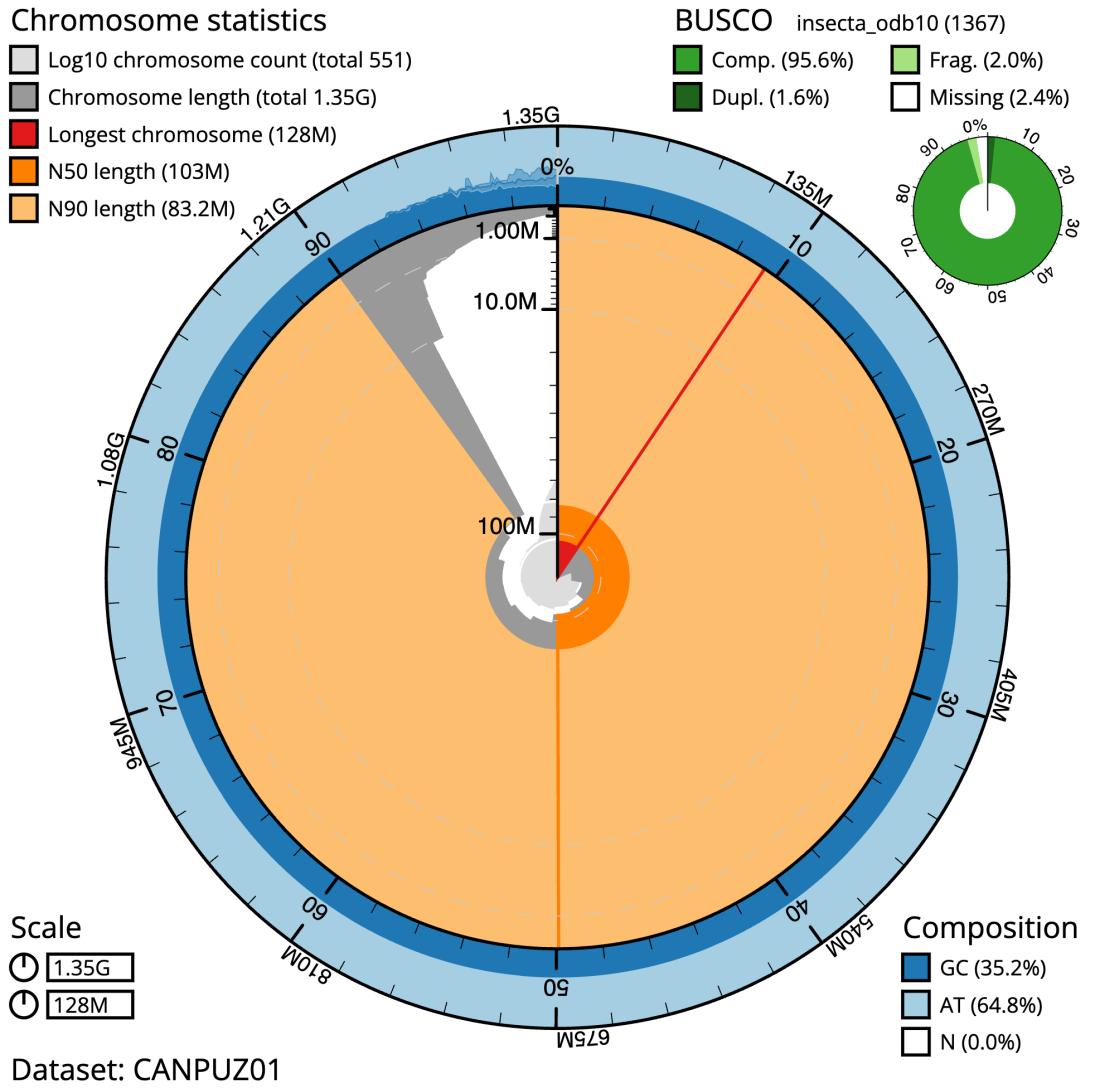
Genome assembly of *Sympetrum striolatum*, ioSymStri1.1: metrics. The BlobToolKit Snailplot shows N50 metrics and BUSCO gene completeness. The main plot is divided into 1,000 size-ordered bins around the circumference with each bin representing 0.1% of the 1,349,614,149 bp assembly. The distribution of scaffold lengths is shown in dark grey with the plot radius scaled to the longest scaffold present in the assembly (128,156,324 bp, shown in red). Orange and pale-orange arcs show the N50 and N90 scaffold lengths (103,201,359 and 83,206,058 bp), respectively. The pale grey spiral shows the cumulative scaffold count on a log scale with white scale lines showing successive orders of magnitude. The blue and pale-blue area around the outside of the plot shows the distribution of GC, AT and N percentages in the same bins as the inner plot. A summary of complete, fragmented, duplicated and missing BUSCO genes in the insecta_odb10 set is shown in the top right. An interactive version of this figure is available at https://blobtoolkit.genomehubs.org/view/ioSymStri1.1/dataset/CANPUZ01/snail.

The final assembly has a total length of 1349.6 Mb in 550 sequence scaffolds with a scaffold N50 of 103.2 Mb ( Table 1). Most (94.4%) of the assembly sequence was assigned to 12 chromosomal-level scaffolds, representing 11autosomes and the X sex chromosome. Chromosome-scale scaffolds confirmed by the Hi-C data are named in order of size ( Figure 2– Figure 5; Table 2). The telomeric satellite repeat number is an estimate. While not fully phased, the assembly deposited is of one haplotype. Contigs corresponding to the second haplotype have also been deposited. The mitochondrial genome was also assembled and can be found as a contig within the multifasta file of the genome submission.

**Table 1.** Genome data for *Sympetrum striolatum*, ioSymStri1.1.

Project accession data		
Assembly identifier	ioSymStri1.1	
Species	*Sympetrum striolatum*	
Specimen	ioSymStri1	
NCBI taxonomy ID	6969	
BioProject	PRJEB55606	
BioSample ID	SAMEA7520376	
Isolate information	ioSymStri1, female: head and thorax (DNA sequencing) ioSymStri2: abdomen (RNA sequencing) ioSymStri3, female: head (Hi-C scaffolding)	
Assembly metrics *		*Benchmark*
Consensus quality (QV)	62.4	*≥ 50*
*k*-mer completeness	100%	*≥ 95%*
BUSCO **	C:95.6%[S:94.0%,D:1.6%],F:2.0%,M:2.4%,n:1,367	*C ≥ 95%*
Percentage of assembly mapped to chromosomes	94.4%	*≥ 95%*
Sex chromosomes	X chromosome	*localised homologous pairs*
Organelles	Mitochondrial genome assembled	*complete single alleles*
Raw data accessions		
PacificBiosciences SEQUEL II	ERR10144336, ERR10123274, ERR10144335	
10X Genomics Illumina	ERR10123722, ERR10123724, ERR10123723, ERR10123725	
Hi-C Illumina	ERR10123726	
PolyA RNA-Seq Illumina	ERR10378028	
Genome assembly		
Assembly accession	GCA_947579665.1	
*Accession of alternate haplotype*	GCA_947579545.1	
Span (Mb)	1349.6	
Number of contigs	900	
Contig N50 length (Mb)	6.1	
Number of scaffolds	550	
Scaffold N50 length (Mb)	103.2	
Longest scaffold (Mb)	128.1	

* Assembly metric benchmarks are adapted from column VGP-2020 of “Table 1: Proposed standards and metrics for defining genome assembly quality” from ( Rhie *et al.*, 2021). ** BUSCO scores based on the insecta_odb10 BUSCO set using v5.3.2. C = complete [S = single copy, D = duplicated], F = fragmented, M = missing, n = number of orthologues in comparison. A full set of BUSCO scores is available at https://blobtoolkit.genomehubs.org/view/ioSymStri1.1/dataset/CANPUZ01/busco.

**Figure 3.**
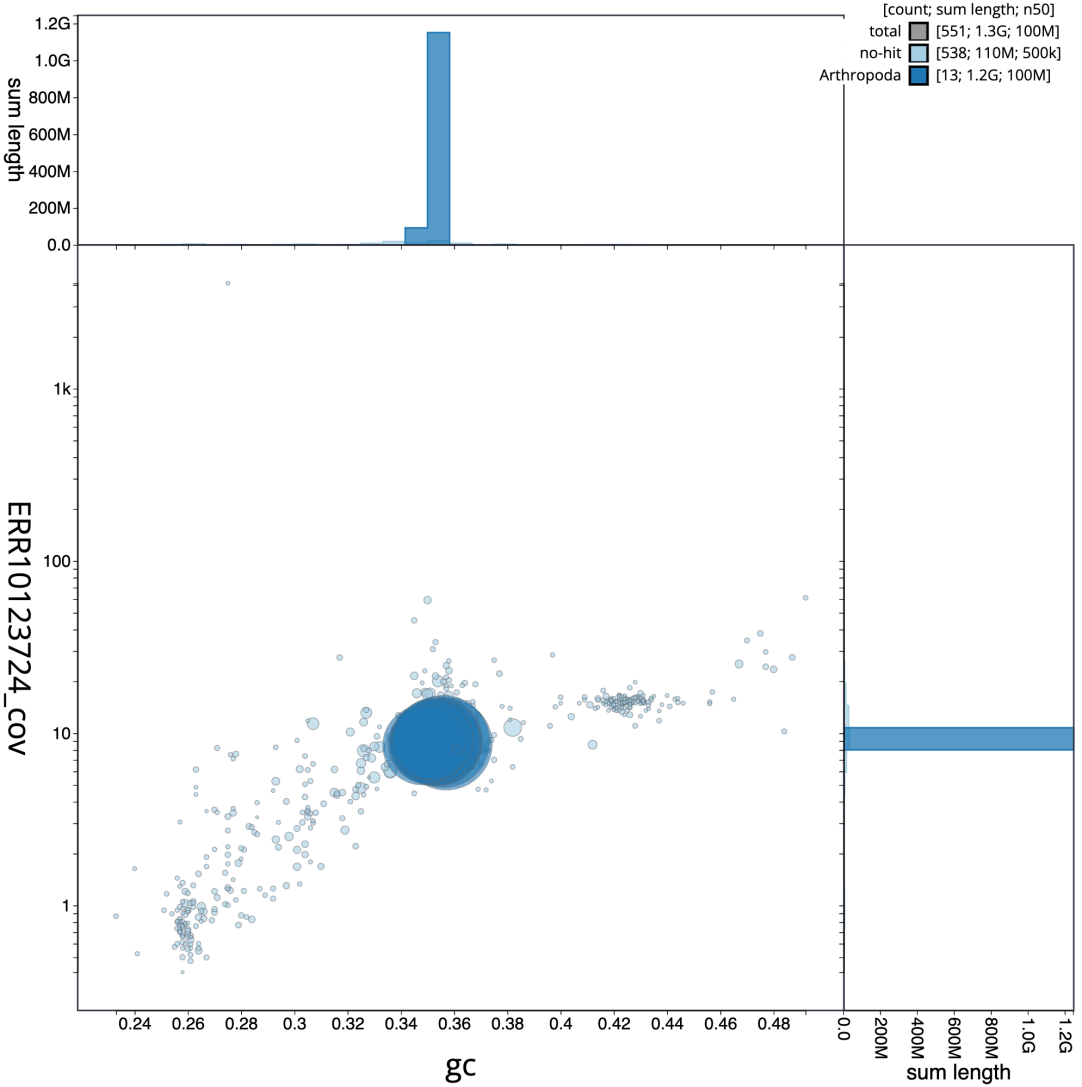
Genome assembly of *Sympetrum striolatum*, ioSymStri1.1: BlobToolKit GC-coverage plot. Scaffolds are coloured by phylum. Circles are sized in proportion to scaffold length. Histograms show the distribution of scaffold length sum along each axis. An interactive version of this figure is available at https://blobtoolkit.genomehubs.org/view/ioSymStri1.1/dataset/CANPUZ01/blob.

**Figure 4.**
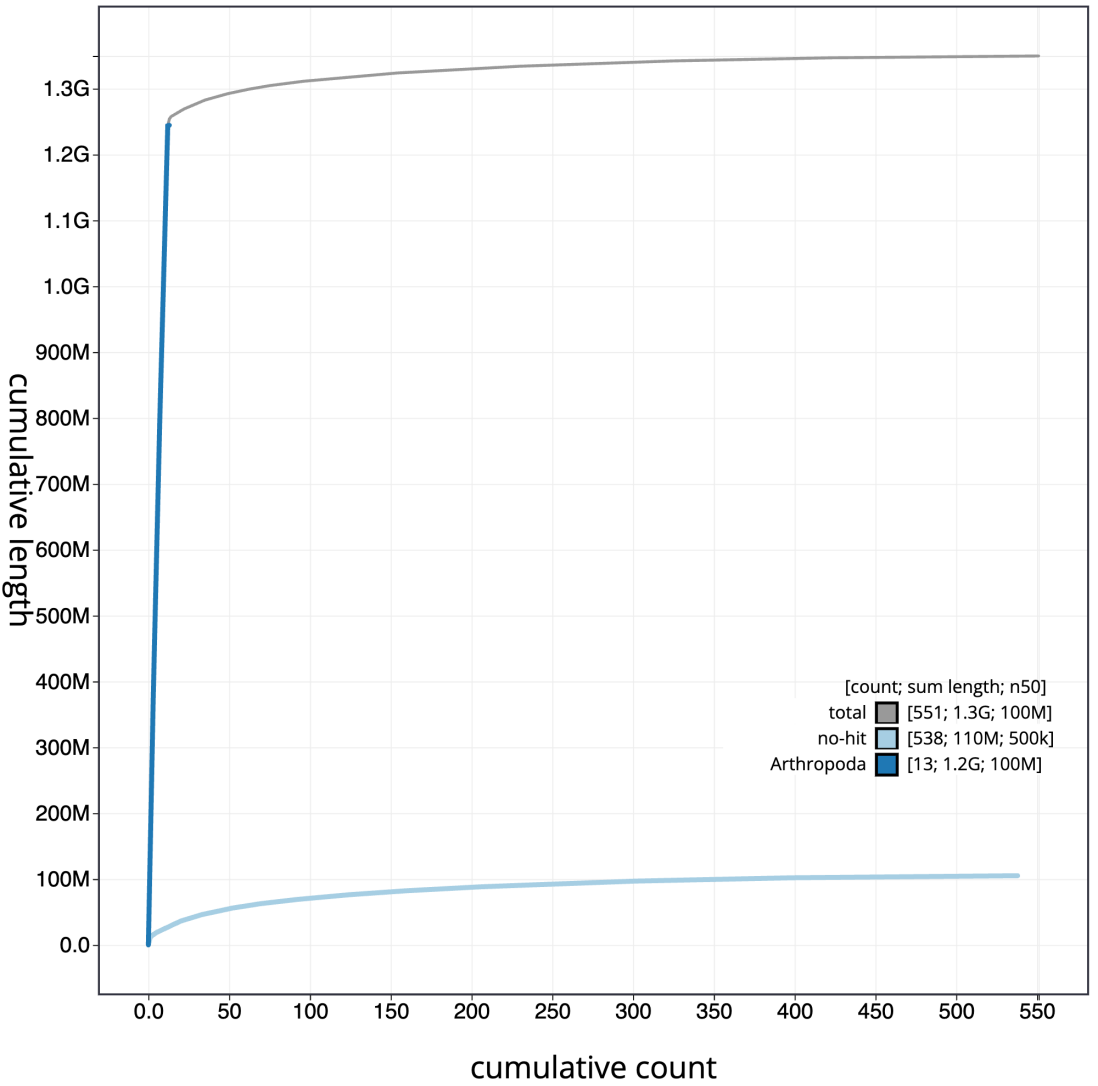
Genome assembly of *Sympetrum striolatum*, ioSymStri1.1: BlobToolKit cumulative sequence plot. The grey line shows cumulative length for all scaffolds. Coloured lines show cumulative lengths of scaffolds assigned to each phylum using the buscogenes taxrule. An interactive version of this figure is available at https://blobtoolkit.genomehubs.org/view/ioSymStri1.1/dataset/CANPUZ01/cumulative.

**Figure 5.**
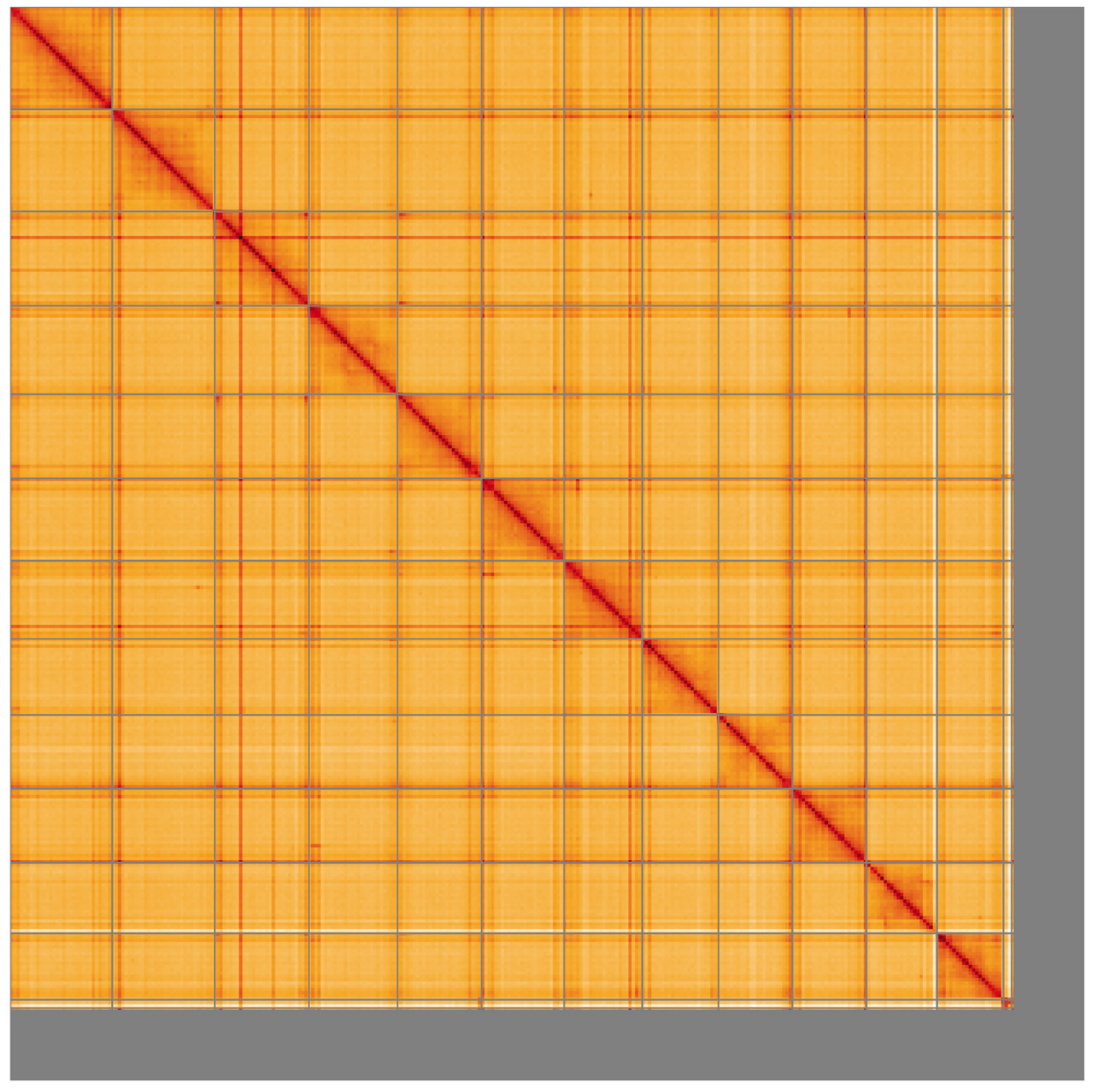
Genome assembly of *Sympetrum striolatum*, ioSymStri1.1: Hi-C contact map of the ioSymStri1.1 assembly, visualised using HiGlass. Chromosomes are shown in order of size from left to right and top to bottom. An interactive version of this figure may be viewed at https://genome-note-higlass.tol.sanger.ac.uk/l/?d=Kh-SUZlORPaevZpr9bP4ag.

**Table 2.** Chromosomal pseudomolecules in the genome assembly of *Sympetrum striolatum*, ioSymStri1.

INSDC accession	Chromosome	Length (Mb)	GC%
OX388341.1	1	128.14	35.5
OX388342.1	2	128.16	35.5
OX388343.1	3	118.11	35.5
OX388344.1	4	110.82	35.5
OX388345.1	5	105.31	35.0
OX388346.1	6	95.12	35.0
OX388347.1	7	103.2	35.5
OX388348.1	8	92.68	35.0
OX388349.1	9	88.51	35.0
OX388350.1	10	92.4	35.0
OX388351.1	11	83.21	35.0
OX388352.1	X	98.05	35.0
OX388353.1	MT	0.02	27.5

The estimated Quality Value (QV) of the final assembly is 62.4 with *k*-mer completeness of 100%, and the assembly has a BUSCO v5.3.2 completeness of 95.6% (single = 94.0%, duplicated = 1.6%), using the insecta_odb10 reference set ( *n* = 1,367).

Metadata for specimens, spectral estimates, sequencing runs, contaminants and pre-curation assembly statistics can be found at https://links.tol.sanger.ac.uk/species/6969.

## Methods

### Sample acquisition and nucleic acid extraction

The specimen used for DNA sequencing was a female *Sympetrum striolatum* (specimen ID Ox000162, ToLID ioSymStri1), which was netted in Wytham Great Wood, Oxfordshire (biological vice-country Berkshire), UK (latitude 51.77, longitude –1.33) on 2019-08-13. The specimen was collected and identified by Liam Crowley (University of Oxford) and was preserved on dry ice.

Two further specimens were netted at Hever Castle, Kent, UK (latitude 51.19, longitude 0.12) on 2020-08-27. The specimens were collected and identified by Benjamin Price and Louise Allan (both Natural History Museum), and were then preserved in liquid nitrogen. The specimen used for RNA sequencing had specimen ID NHMUK014444679 (ToLID ioSymStri2), while the specimen used for Hi-C data had specimen ID NHMUK014444680, (ToLID ioSymStri3).

DNA was extracted at the Tree of Life laboratory, Wellcome Sanger Institute (WSI). The ioSymStri1 sample was weighed and dissected on dry ice. Head and thorax tissue was disrupted using a Nippi Powermasher fitted with a BioMasher pestle *.* High molecular weight (HMW) DNA was extracted using the Qiagen MagAttract HMW DNA extraction kit. Low molecular weight DNA was removed from a 20 ng aliquot of extracted DNA using the 0.8X AMpure XP purification kit prior to 10X Chromium sequencing; a minimum of 50 ng DNA was submitted for 10X sequencing. HMW DNA was sheared into an average fragment size of 12–20 kb in a Megaruptor 3 system with speed setting 30. Sheared DNA was purified by solid-phase reversible immobilisation using AMPure PB beads with a 1.8X ratio of beads to sample to remove the shorter fragments and concentrate the DNA sample. The concentration of the sheared and purified DNA was assessed using a Nanodrop spectrophotometer and Qubit Fluorometer and Qubit dsDNA High Sensitivity Assay kit. Fragment size distribution was evaluated by running the sample on the FemtoPulse system.

RNA was extracted from abdomen tissue of ioSymStri2 in the Tree of Life Laboratory at the WSI using TRIzol, according to the manufacturer’s instructions. RNA was then eluted in 50 μl RNAse-free water and its concentration assessed using a Nanodrop spectrophotometer and Qubit Fluorometer using the Qubit RNA Broad-Range (BR) Assay kit. Analysis of the integrity of the RNA was done using Agilent RNA 6000 Pico Kit and Eukaryotic Total RNA assay.

### Sequencing

Pacific Biosciences HiFi circular consensus and 10X Genomics read cloud DNA sequencing libraries were constructed according to the manufacturers’ instructions. Poly(A) RNA-Seq libraries were constructed using the NEB Ultra II RNA Library Prep kit. DNA and RNA sequencing was performed by the Scientific Operations core at the WSI on Pacific Biosciences SEQUEL II (HiFi), Illumina NovaSeq 6000 (RNA-Seq) and HiSeq X Ten (10X) instruments. Hi-C data were also generated from head tissue of ioSymStri3 using the Arima2 kit and sequenced on the Illumina NovaSeq 6000 instrument.

### Genome assembly, curation and evaluation

Assembly was carried out with Hifiasm ( Cheng *et al.*, 2021) and haplotypic duplication was identified and removed with purge_dups ( Guan *et al.*, 2020). One round of polishing was performed by aligning 10X Genomics read data to the assembly with Long Ranger ALIGN, calling variants with FreeBayes ( Garrison & Marth, 2012). The assembly was then scaffolded with Hi-C data ( Rao *et al.*, 2014) using YaHS ( Zhou *et al.*, 2023). The assembly was checked for contamination and corrected using the gEVAL system ( Chow *et al.*, 2016) as described previously ( Howe *et al.*, 2021). Manual curation was performed using gEVAL, HiGlass ( Kerpedjiev *et al.*, 2018) and Pretext ( Harry, 2022). The mitochondrial genome was assembled using MitoHiFi ( Uliano-Silva *et al.*, 2022), which runs MitoFinder ( Allio *et al.*, 2020) or MITOS ( Bernt *et al.*, 2013) and uses these annotations to select the final mitochondrial contig and to ensure the general quality of the sequence.

A Hi-C map for the final assembly was produced using bwa-mem2 ( Vasimuddin *et al.*, 2019) in the Cooler file format ( Abdennur & Mirny, 2020). To assess the assembly metrics, the *k*-mer completeness and QV consensus quality values were calculated in Merqury ( Rhie *et al.*, 2020). This work was done using Nextflow ( Di Tommaso *et al.*, 2017) DSL2 pipelines “sanger-tol/readmapping” ( Surana *et al.*, 2023a) and “sanger-tol/genomenote” ( Surana *et al.*, 2023b). The genome was analysed within the BlobToolKit environment ( Challis *et al.*, 2020) and BUSCO scores ( Manni *et al.*, 2021; Simão *et al.*, 2015) were calculated.

Table 3 contains a list of relevant software tool versions and sources.

**Table 3.** Software tools: versions and sources.

Software tool	Version	Source
BlobToolKit	4.0.7	https://github.com/blobtoolkit/blobtoolkit
BUSCO	5.3.2	https://gitlab.com/ezlab/busco
FreeBayes	1.3.1-17-gaa2ace8	https://github.com/freebayes/freebayes
gEVAL	N/A	https://geval.org.uk/
Hifiasm	0.16.1-r375	https://github.com/chhylp123/hifiasm
HiGlass	1.11.6	https://github.com/higlass/higlass
Long Ranger ALIGN	2.2.2	https://support.10xgenomics.com/genome-exome/software/pipelines/latest/advanced/other-pipelines
Merqury	MerquryFK	https://github.com/thegenemyers/MERQURY.FK
MitoHiFi	2	https://github.com/marcelauliano/MitoHiFi
PretextView	0.2	https://github.com/wtsi-hpag/PretextView
purge_dups	1.2.3	https://github.com/dfguan/purge_dups
sanger-tol/genomenote	v1.0	https://github.com/sanger-tol/genomenote
sanger-tol/readmapping	1.1.0	https://github.com/sanger-tol/readmapping/tree/1.1.0
YaHS	yahs-1.1.91eebc2	https://github.com/c-zhou/yahs

### Wellcome Sanger Institute – Legal and Governance

The materials that have contributed to this genome note have been supplied by a Darwin Tree of Life Partner. The submission of materials by a Darwin Tree of Life Partner is subject to the **‘Darwin Tree of Life Project Sampling Code of Practice’**, which can be found in full on the Darwin Tree of Life website here. By agreeing with and signing up to the Sampling Code of Practice, the Darwin Tree of Life Partner agrees they will meet the legal and ethical requirements and standards set out within this document in respect of all samples acquired for, and supplied to, the Darwin Tree of Life Project.

Further, the Wellcome Sanger Institute employs a process whereby due diligence is carried out proportionate to the nature of the materials themselves, and the circumstances under which they have been/are to be collected and provided for use. The purpose of this is to address and mitigate any potential legal and/or ethical implications of receipt and use of the materials as part of the research project, and to ensure that in doing so we align with best practice wherever possible. The overarching areas of consideration are:

•   Ethical review of provenance and sourcing of the material

•   Legality of collection, transfer and use (national and international) 

Each transfer of samples is further undertaken according to a Research Collaboration Agreement or Material Transfer Agreement entered into by the Darwin Tree of Life Partner, Genome Research Limited (operating as the Wellcome Sanger Institute), and in some circumstances other Darwin Tree of Life collaborators.

## Data Availability

European Nucleotide Archive: S *ympetrum striolatum* (common darter). Accession number PRJEB55606; https://identifiers.org/ena.embl/PRJEB55606. ( Wellcome Sanger Institute, 2022) The genome sequence is released openly for reuse. The *Sympetrum striolatum* genome sequencing initiative is part of the Darwin Tree of Life (DToL) project. All raw sequence data and the assembly have been deposited in INSDC databases. The genome will be annotated using available RNA-Seq data and presented through the Ensembl pipeline at the European Bioinformatics Institute. Raw data and assembly accession identifiers are reported in Table 1.

## Author information

Members of the University of Oxford and Wytham Woods Genome Acquisition Lab are listed here: https://doi.org/10.5281/zenodo.4789928.

Members of the Natural History Museum Genome Acquisition Lab are listed here: https://doi.org/10.5281/zenodo.4790042.

Members of the Darwin Tree of Life Barcoding collective are listed here: https://doi.org/10.5281/zenodo.4893703.

Members of the Wellcome Sanger Institute Tree of Life programme are listed here: https://doi.org/10.5281/zenodo.4783585.

Members of Wellcome Sanger Institute Scientific Operations: DNA Pipelines collective are listed here: https://doi.org/10.5281/zenodo.4790455.

Members of the Tree of Life Core Informatics collective are listed here: https://doi.org/10.5281/zenodo.5013541.

Members of the Darwin Tree of Life Consortium are listed here: https://doi.org/10.5281/zenodo.4783558.
