# Supplementary material for: The genome sequence of the Common Darter, Sympetrum striolatum (Charpentier, 1840)
Source: Wellcome Open Res. 2023 Sep 1;8:389. [Version 1] doi: 10.12688/wellcomeopenres.19937.1 (PMC10904951; doi:10.12688/wellcomeopenres.19937.1)
Supplement: Peer review history

# Reviewer report: Reviewer response for version 1

**DOI:** 10.21956/wellcomeopenres.22077.r72217

**Published:** 2024-02-19

**Authors:** Panagiotis Ioannidis

**Affiliations:** 1 Foundation for Research & Technology Hellas, Crete, Greece

The manuscript by Crowley *et al.* describes the sampling, sequencing, and assembly of the genome of the odonate species Sympetrum striolatum. I believe that adding one more odonate genome to the public domain is very important because of the ecological importance of this "genomically neglected" insect clade.

 The genome is of high quality. First, its contiguity is high since almost all of its sequence is found in just 12 chromosome-level scaffolds. Second, its BUSCO scores are also high with 95.6% complete BUSCOs, of which only 1.6% was found as duplicated.

 The authors also include the results of other analyses as well which add value to this genome report and will definitely help the relevant research community.

 As a bottom line, I fully endorse this manuscript for indexing in its current form.

Are sufficient details of methods and materials provided to allow replication by others?

Yes

Is the rationale for creating the dataset(s) clearly described?

Yes

Are the datasets clearly presented in a useable and accessible format?

Yes

Are the protocols appropriate and is the work technically sound?

Yes

Reviewer Expertise:

Insect genomics and transcriptomics

I confirm that I have read this submission and believe that I have an appropriate level of expertise to confirm that it is of an acceptable scientific standard.

# Reviewer report: Reviewer response for version 1

**DOI:** 10.21956/wellcomeopenres.22077.r72349

**Published:** 2024-02-13

**Authors:** Jerome Hui

**Affiliations:** 1 The Chinese University of Hong Kong, Hong Kong, China

Crowley and colleagues report the genome sequence of a female dragonfly *Sympetrum striolatum *(Charpentier, 1840). This species is abundant across the British Isles. Prior to this report, molecular data of this species are mainly confined to ribosomal, ITS, and mitochondrion sequences deposited to the NCBI database. This new genome resource will be useful for further studies, including (but not limited to) understanding their population structure, whether there could be subspecies, evolutionary relationships with other dragonflies, as well as effects of climate change on them.

 This genome resource is excellent from the summary statistics, with high BUSCO numbers, high sequence continuity (scaffold N50), and majority of sequences contained on the 12 pseudochromosomes (including sex chromosome X) plus mitochondrion. To sum up, this is a valuable contribution.

Are sufficient details of methods and materials provided to allow replication by others?

Yes

Is the rationale for creating the dataset(s) clearly described?

Yes

Are the datasets clearly presented in a useable and accessible format?

Yes

Are the protocols appropriate and is the work technically sound?

Yes

Reviewer Expertise:

Genomics, evolution, invertebrates

I confirm that I have read this submission and believe that I have an appropriate level of expertise to confirm that it is of an acceptable scientific standard.

# Reviewer report: Reviewer response for version 1

**DOI:** 10.21956/wellcomeopenres.22077.r69721

**Published:** 2024-01-19

**Authors:** Manpreet Kohli

**Affiliations:** 1 Department of Natural Sciences, Baruch College, City University of New York, New York, New York, USA; 2 Division of Invertebrate Zoology, American Museum of Natural History, New York, New York, USA

This article presents the whole genome sequencing for the dragonfly species, *Sympetrum striolatum*. Authors have used cutting edge technology to sequence then whole genome and "seemingly" also the transcriptome of this species of dragonfly. I am unsure about the transcriptomes, because the authors mention sequencing the RNA from the sample isoSymStri2 but don't actually discuss the results of RNA sequencing or how it was further used in any analysis to better inform the assembly or characteristics of the genome. The methods used by the authors in their the genome assembly pipeline are upto the standard of this field. 

 While authors have provided a good introduction to this species, they have not sufficiently eluded to why we need to sequence the genome for this species. I think the manuscript will benefit from a brief discussion of how genomes sequence from this species can further our understanding of the evolution of dragonflies.

Are sufficient details of methods and materials provided to allow replication by others?

Yes

Is the rationale for creating the dataset(s) clearly described?

Partly

Are the datasets clearly presented in a useable and accessible format?

Yes

Are the protocols appropriate and is the work technically sound?

Yes

Reviewer Expertise:

Genomics, Insect Evolution

I confirm that I have read this submission and believe that I have an appropriate level of expertise to confirm that it is of an acceptable scientific standard, however I have significant reservations, as outlined above.
